# Evaluating Triage Acuity and Injury Mechanisms as Drivers of Emergency Department Length of Stay in Pediatric Forensic Cases

**DOI:** 10.3390/jcm15187110

**Published:** 2026-09-13

**Authors:** Muhammet Raşit Özer, Ömer Jaradat, Kadir Küçükceran, Ali Avcı

**Affiliations:** 1 Faculty of Medicine, Department of Emergency Medicine, Karamanoğlu Mehmetbey University, 70100 Karaman, Türkiye; drrasitozer@gmail.com; 2 Faculty of Medicine, Department of Emergency Medicine, Necmettin Erbakan University, 42080 Konya, Türkiye; kadirkucukceran@hotmail.com; 3 Department of Emergency Medicine, Karaman Training and Research Hospital, 70200 Karaman, Türkiye; aliavci171@hotmail.com

**Keywords:** pediatrics, emergency medicine, forensic cases, length of stay, triage, Emergency Severity Index

## Abstract

**Background/Objectives**: Pediatric forensic cases represent a growing public health concern and impose substantial burdens on emergency departments (EDs). However, data on operational predictors of ED length of stay (ED-LOS) in this specific population remain limited. We aimed to identify predictors of increased ED length of stay (ED-LOS) and evaluate clinical profiles of pediatric forensic cases. **Methods**: This retrospective observational study included 1113 pediatric forensic cases. Cox proportional hazards regression was used as the primary method to identify predictors of time-to-discharge. Firth’s penalized logistic regression and Random Forest (RF) were used for binary prediction based on a data-derived 45 min high-acuity threshold established via Reciever Operating Characteristic (ROC) analysis. Performance was assessed via AUC, Brier score, bootstrap validation, and Decision Curve Analysis (DCA). **Results**: The cohort was predominantly adolescent (71.5%) and male (65.3%), with interpersonal assault (36.7%) and standard forensic examinations (26.7%) being the most frequent etiologies. In the Cox regression, lower clinical acuity (higher Emergency Severity Index [ESI] scores) significantly increased the hazard of discharge (Hazard Ratio [HR]: 1.954). Conversely, specific trauma mechanisms notably delayed discharge, particularly intoxication (HR: 0.126), burns (HR: 0.127), and out-of-vehicle traffic accidents (HR: 0.237). Parent-accompanied presentation was independently associated with faster ED processing (HR: 1.303), while ambulance arrival was not a significant independent driver of overall time-to-discharge (*p* = 0.176). RF confirmed that the ESI was the dominant operational predictor (37.0% importance; test AUC 0.906), and the Firth regression demonstrated that these same mechanisms were the strongest predictors. The model showed high Negative Predictive Value (96.9%) and DCA benefit. **Conclusions**: This study proposes a framework for predicting increased ED-LOS in pediatric forensic cases. Cox regression suggested that ESI and trauma mechanisms modulate discharge speed, reflecting operational patterns. The findings may support triage stratification and resource allocation, but external validation is needed.

## 1. Introduction

In recent years, there has been a significant global increase in childhood injuries, poisonings, and maltreatment cases [1]. According to the World Health Organization (WHO), approximately 1 billion children aged 2–17 years are estimated to have experienced physical, emotional, or sexual violence [1,2,3]. These injuries account for approximately 40% of all childhood deaths worldwide [1,4,5]. In Turkey, the majority of deaths among children aged 1–17 years have been reported to result from external injuries and poisonings [4]. Assault, torture, traffic accidents, firearm injuries, occupational accidents, poisonings, burns, sexual assault and abuse, suicide attempts, and other accidents in childhood are classified as forensic cases under the Turkish Penal Code [6]. Pediatric forensic cases continue to increase over time, representing a serious public health concern and imposing a substantial burden on the healthcare system [7,8].

Furthermore, defining pediatric forensic cases presenting to the Emergency Department (ED) based on age, sex, and diagnosis, and developing targeted clinical approaches toward common forensic diagnoses, is of great importance for effective intervention planning [8]. The extreme crowding in EDs, compounded by the additional workload imposed by forensic processes, necessitates the accurate and rapid determination of clinical urgency in this vulnerable patient population. Triage systems are vital tools that classify the urgency of patients’ medical needs according to objective criteria, enabling efficient resource utilization. The five-level Emergency Severity Index (ESI), which is a validated triage instrument, is widely used in the pediatric population, as it simultaneously assesses both clinical severity and estimated resource needs [9]. In the management of pediatric forensic cases in the ED, analyzing clinical urgency levels alongside the temporal dynamics introduced by forensic processes (such as ED Length of Stay [ED-LOS]) is of critical clinical importance for improving the quality of medical care and ensuring timely forensic reporting [1].

Despite the increasing burden of pediatric forensic cases and the growing body of descriptive epidemiological literature, there is a lack of data regarding operational predictors of ED-LOS in this specific population [1,6,8]. Most existing studies have focused on demographic profiling, injury patterns, and seasonal variations, with limited attention given to predictive modeling that could guide real-time triage and resource allocation. To our knowledge, this is the first study to identify independent predictors of ED-LOS in pediatric forensic cases. Therefore, this study aimed to identify specific trauma mechanisms that significantly delay ED discharge, quantify the relative importance of triage acuity versus injury mechanism in predicting prolonged ED-LOS, and demonstrate the clinical utility of these findings via Decision Curve Analysis (DCA), with the goal of supporting triage efficiency, resource allocation, and workflow optimization in pediatric forensic emergency settings.

## 2. Materials and Methods

### 2.1. Study Design and Participants

This observational, retrospective study was conducted at the ED of Karaman Training and Research Hospital. The study analyzed data from patients admitted to the ED between 1 January 2022 and 31 December 2022. The data extraction and retrospective statistical analyses were subsequently conducted between November 2023 and March 2024. The study site is the sole state hospital and the only tertiary care center (Level 1 trauma center) in the central district of Karaman, with an average monthly volume of approximately 1000 forensic case presentations. The study commenced following approval by the Karamanoğlu Mehmetbey University Medical Faculty Ethics Committee (Approval No.: E-11095095-050.01.04-154196; date: 5 October 2023). The study was conducted in accordance with the principles of the Declaration of Helsinki. Due to the retrospective design, informed consent from patients or their legal guardians was waived. Reporting adheres to the STROBE statement for observational studies, with the items pertinent to model performance reported according to the TRIPOD statement where applicable [10,11].

### 2.2. Study Population

All pediatric forensic cases aged <18 years admitted to the ED trauma unit were included. Eligible cases comprised those presenting due to forensic examination, traffic accidents (in-vehicle and out-of-vehicle), falls, penetrating injuries (sharp and firearm), occupational accidents, intoxication, burns, sexual assault/abuse, or suicide attempts. Cases not evaluated as forensic were excluded from the study. Participants were stratified into four developmental periods for analysis: infancy (0–2 years), early childhood (3–6 years), middle childhood (7–11 years), and adolescence (12–18 years).

### 2.3. Study Protocol and Data Collection

Patient data were retrieved from the hospital’s electronic medical records and patient files. Demographic variables, including age and sex, as well as admission dates and discharge diagnoses, were recorded. Forensic cases were categorized according to International Classification of Diseases codes into the following groups: forensic examination, in-vehicle and out-of-vehicle traffic accidents, penetrating injuries (sharp object and firearm), assault, sexual abuse, falls, burns, occupational accidents, suicide attempts, and intoxication.

Standard forensic examinations were defined as medico-legal evaluations performed for documentation purposes in cases without acute traumatic injuries requiring immediate intervention. In Turkey, these examinations are mandated by the judicial system and may be requested by law enforcement before or after police custody (custodial entry/exit examinations), by court order for evidence collection, or by social services for pre-placement health screenings. This subgroup includes pediatric patients brought to the ED for compulsory legal documentation, often without active medical complaints. Typical procedures include routine physical examination to document signs of trauma, court-ordered examinations for suspected neglect, and abuse. The resulting medico-legal reports serve to guide judicial authorities in establishing the factual basis of cases involving children [12,13,14].

To determine the clinical urgency of all included patients, triage records from the hospital automation system were retrospectively reviewed. Based on the patients’ clinical status and resource needs at initial ED admission, the validated five-level ESI version 4 algorithm was utilized to assign ESI scores (ranging from ESI-1: most urgent/resuscitation to ESI-5: least urgent) [15]. Accordingly, patients were further classified into triage zones: Red (ESI-1 and ESI-2), Yellow (ESI-3), and Green (ESI-4 and ESI-5).

The following variables were extracted and analyzed in detail: ED length of stay (ED-LOS, in minutes), mode of presentation (ambulance, self-presentation, parent-accompanied, or other), and patient disposition (discharge, hospitalization, transfer to another facility, or mortality).

### 2.4. Outcomes

The primary outcome of this study was the ED-LOS, defined as the continuous time interval (in minutes) from initial patient registration at the ED triage desk to the completion of the ED episode. Secondary outcomes included assessing the relationship between ESI triage acuity and ED operational flow, evaluating the demographic profiles, etiological characteristics, and overall clinical dispositions of forensic pediatric patients. Collectively, these secondary objectives seek to establish a regional epidemiological baseline and guide proactive resource allocation to prevent delays in acute medical interventions.

### 2.5. Statistical Analyses

All statistical analyses, predictive modeling, and advanced visualizations were performed using a programmatic computational pipeline integrating R software (version 4.3.1; R Foundation for Statistical Computing, Vienna, Austria) and Python (version 3.10; Python Software Foundation, Wilmington, DE, USA) within a cloud-based Jupyter notebook environment (Google Colaboratory; Google LLC, Mountain View, CA, USA). The logistf package (version 1.26.1) in R was utilized for the primary Firth’s penalized-likelihood logistic regression models. In Python, data processing, statistical computations, and performance metric evaluations were executed using the pandas (version 2.0.3), NumPy (version 1.25.2), SciPy (version 1.11.4), and scikit-learn (version 1.2.2) libraries. DCA was programmatically constructed and visualized utilizing the matplotlib (version 3.7.1) library. A two-sided *p*-value < 0.05 was considered statistically significant for all analyses.

#### 2.5.1. Descriptive and Comparative Statistics

Data distribution was assessed using histograms and the Kolmogorov–Smirnov test. Normally distributed continuous variables (age by sex) were compared using the Independent-Samples T-Test and presented as means ± standard deviation (SD). Non-normally distributed variables (ESI scores, ED-LOS) were reported as medians with interquartile ranges (IQR: 25th–75th percentiles) and analyzed using Mann–Whitney U or Kruskal–Wallis tests. Categorical variables were compared using the Chi-square test, with post-hoc analyses conducted for multiple group comparisons.

#### 2.5.2. Cox Proportional Hazards Regression

To establish the operational impact of predictors without the bias of dichotomization, we primarily employed a continuous-time Cox proportional hazards regression as our core analytical strategy. This approach analyzed ED-LOS as a continuous time variable (minutes from registration to discharge), with all patients considered uncensored as they completed their ED episode. The Cox model evaluated the precise duration of stay in minutes, rather than relying on any binary cut-off, making it less susceptible to the mathematical coupling inherent in threshold-based models. The proportional hazards assumption was validated using Schoenfeld residuals and was not violated (*p* > 0.05 for all covariates).

#### 2.5.3. Model Discrimination and Threshold Determination

To translate these continuous-time findings into an actionable operational tool, a data-driven temporal threshold for ED-LOS was established using Reciever Operating Characteristic (ROC) analysis. Model discrimination was evaluated using the Area Under the Receiver Operating Characteristic Curve (AUC). The optimal threshold was established directly from the data by maximizing the Youden Index (J = Sensitivity + Specificity − 1) in an ROC analysis evaluating the prediction of high clinical acuity (ESI scores 1–3). The same Youden Index optimization was utilized to define the optimal risk probability cut-offs for the predictive models, yielding standardized diagnostic metrics including sensitivity, specificity, positive predictive value (PPV), and negative predictive value (NPV).

#### 2.5.4. Firth’s Penalized Logistic Regression

To confirm that the continuous-time findings translate to a clinically intuitive binary rule, univariate and multivariable Firth-penalized logistic regression analyses were performed. Candidate variables for inclusion in the multivariable models were selected a priori based on clinical relevance and evidence from the existing literature, rather than through data-driven selection algorithms. To mitigate the risks of sparse-data bias, complete separation, and inflated standard errors inherent in conventional maximum likelihood estimation, and to safely adhere to events-per-variable constraints, all models were fitted utilizing Firth’s penalized-likelihood logistic regression (via the logistf package [version 1.26.1] in R), as the penalization effectively reduces the bias and variance associated with sparse data [16]. Multicollinearity among independent predictors was ruled out using the Variance Inflation Factor (VIF), with a conservative threshold of VIF < 2.5 confirming the absence of collinearity. Automated stepwise selection algorithms were strictly avoided to prevent post-selection inference bias and parameter instability. The final multivariable models included age, sex, ESI score, forensic case type (11 categories), season, mode of presentation (4 categories), and admission time (4 categories). All candidate variables met the events-per-variable criteria (≥10 events per variable) and were retained in the final models.

#### 2.5.5. Model Calibration, Internal Validation, and Clinical Utility

Model calibration was assessed quantitatively via the Brier score and visually inspected using calibration plots comparing the predicted versus observed probabilities against an ideal 45-degree reference line. The proportion of variance explained by the models was appraised using Nagelkerke’s and McFadden’s pseudo-R2. To ensure internal validity and quantify potential optimism bias, a 500-repetition bootstrap resampling technique was employed to calculate optimism-corrected AUC (C-index) and corrected Brier scores. Finally, the net clinical utility of the predictive models across a continuum of risk thresholds was evaluated using DCA. A two-sided *p*-value < 0.05 was considered statistically significant for all analyses.

#### 2.5.6. Machine Learning Algorithm Development and Validation

To capture potential non-linear interactions, a Random Forrest (RF) classifier was developed using Python’s scikit-learn library. While the primary Firth regression utilized full-cohort bootstrap validation to maximize the statistical power for odds ratio estimation, a split-sample methodology was necessary for unbiased machine learning evaluation. The cohort (N = 1113) was randomly partitioned into a stratified training set (70%, n = 779) and a held-out test set (30%, n = 334). The RF model was trained exclusively on the training data. To prevent overfitting, the hyperparameters were optimized via an automated Grid Search with 5-fold cross-validation. The optimal configuration utilized 100 decision trees, a maximum depth of 15 nodes, and minimum sample splits of 10 for internal nodes and 1 for terminal leaves (random_state = 42). For an equitable performance comparison, the Firth regression baseline was retrained on the 70% training set. Both models were subsequently evaluated on the unseen 30% test set. Differences in AUC discrimination were assessed using the DeLong test. Finally, to ensure clinical interpretability, the relative predictive weight of each independent variable in the RF model was quantified using the mean decrease in Gini impurity.

## 3. Results

### 3.1. Baseline Demographic and Clinical Characteristics

**Figure 1:**
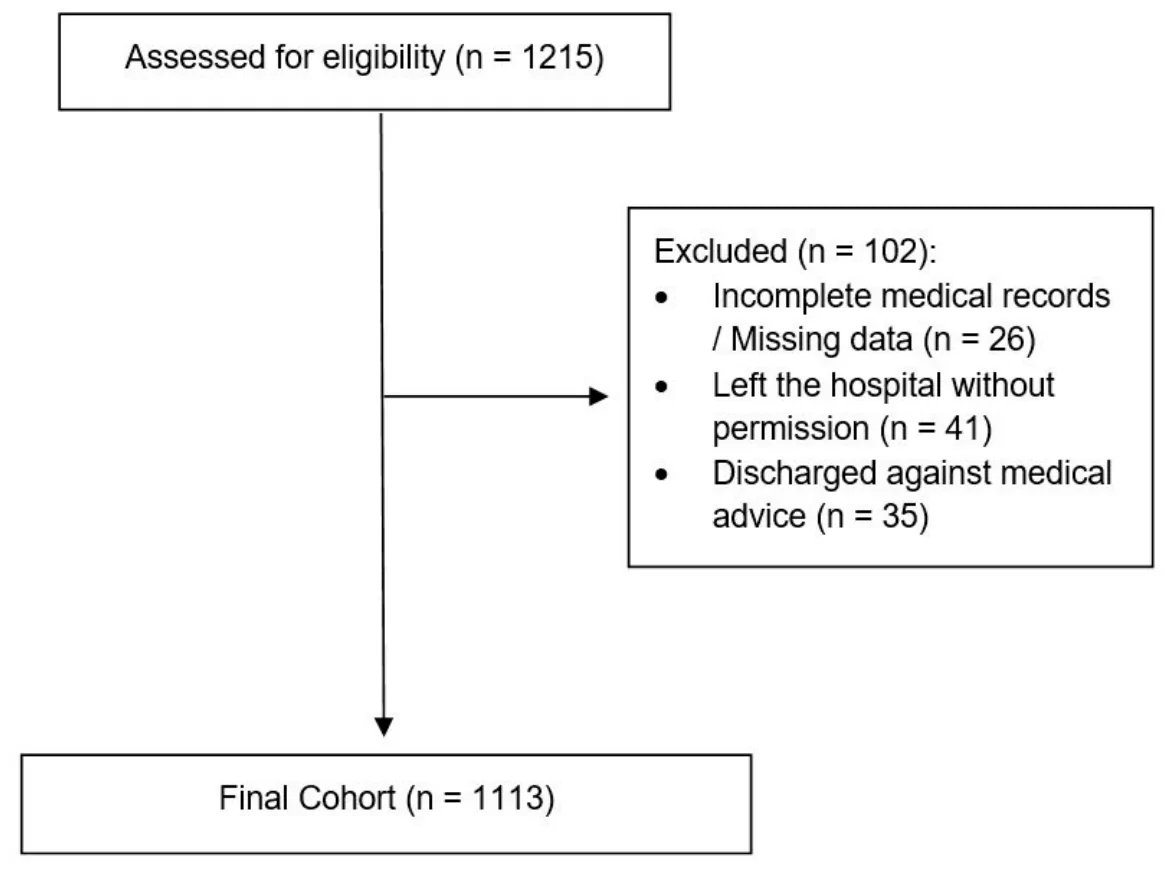
Participant flowchart.

A total of 1215 pediatric forensic cases were initially identified. After excluding 102 patients (26 with missing data, 41 who left without permission, and 35 discharged against medical advice due to parental refusal), 1113 patients were included in the final analysis (Figure 1). The cohort demonstrated a male predominance (65.3%, n = 727), with a median age of 15 years (IQR: 10–16). Adolescents constituted the vast majority of presentations (71.5%), with the male-to-female ratio diverging significantly during this developmental period. Overall, the median ED-LOS was 12.0 min (IQR: 3.0–60.5). Most patients were discharged following outpatient management (90.7%), while 9.3% required hospitalization. A total of eight mortalities were recorded (three in the ED and five during inpatient/Intensive Care Unit care).

Admission volume peaked during the evening shift (15:00–20:59; 39.0%) and summer months (34.0%). According to the ESI algorithm, the vast majority of cases were triaged to the Green zone (83.2%), followed by the Yellow (10.2%) and Red (6.6%) zones. Detailed demographic and clinical characteristics are summarized in Table 1.

### 3.2. Distribution of Forensic Case Types

Assault was the most common forensic presentation, accounting for 36.7% (n = 409) of all cases, followed by standard forensic examinations (26.7%, n = 297), out-of-vehicle traffic accidents (13.6%, n = 151), and in-vehicle traffic accidents (12.1%, n = 135). Adolescence constituted the majority of cases across nearly all categories, with exceptionally high proportions observed for occupational accidents (95.8%), suicide attempts (87.5%), forensic examinations (91.6%), and assault (73.1%). Statistically significant associations with the developmental period were identified for forensic examinations (*p* < 0.001), traffic accidents (*p* < 0.001), assaults (*p* = 0.028), falls (*p* < 0.001), intoxication (*p* < 0.001), and sharp object injuries (*p* = 0.019). Although rare, intoxication cases were notably more frequent during early childhood (44.4%) and infancy (33.3%), while falls demonstrated a bimodal distribution, with peaks in both early childhood and adolescence (Table 2).

As detailed in Tables S1 and S2, a significant association was found between ESI scores and ED-LOS (*p* < 0.001). Patients with ESI-5 (lowest acuity) had the shortest median LOS (3 min; IQR: 2–7), while ESI-3 patients had the longest (132.5 min; IQR: 87.75–225.25). Post-hoc analyses revealed significant differences between ESI-5 and all other ESI groups (*p* < 0.001 for all), whereas no significant differences were observed between the ESI-1, ESI-2, and ESI-3 groups. Similarly, triage zone analysis demonstrated a significant difference in LOS across the three zones (*p* < 0.001). Yellow zone patients had the longest median LOS (132.5 min; IQR: 87.75–225.25), followed by the Red zone (118 min; IQR: 60.5–182.5), while Green zone patients had the shortest (7 min; IQR: 2–30). Pairwise comparisons showed no significant difference between the Red and Yellow zones (*p* > 0.999), but both were significantly longer than the Green zone (*p* < 0.001 for both).

### 3.3. Cox Proportional Hazards Regression for ED-LOS as a Continuous Time Variable

The continuous time Cox proportional hazards regression results are summarized in Table 3. Consistent with the binary logistic models, higher ESI scores (indicating lower clinical acuity) (HR: 1.954, 95% CI: 1.771–2.155, *p* < 0.001) and advancing patient age (HR: 1.022, 95% CI: 1.007–1.037, *p* = 0.004) were independently associated with a significantly increased hazard of discharge, confirming faster ED processing for these demographics.

Conversely, all specific forensic mechanisms significantly reduced the hazard of discharge compared to standard forensic examinations. The most pronounced delays were inherently driven by intoxication, burns, and out-of-vehicle traffic accidents. Regarding the mode of presentation, parent-accompanied arrivals were discharged significantly faster, corroborating the protective effect observed previously. However, ambulance arrival was not significantly associated with the overall continuous time-to-discharge trajectory (HR: 0.879, 95% CI: 0.729–1.059, *p* = 0.176).

### 3.4. Kaplan–Meier Survival Analysis by ESI Triage Zones

**Figure 2:**
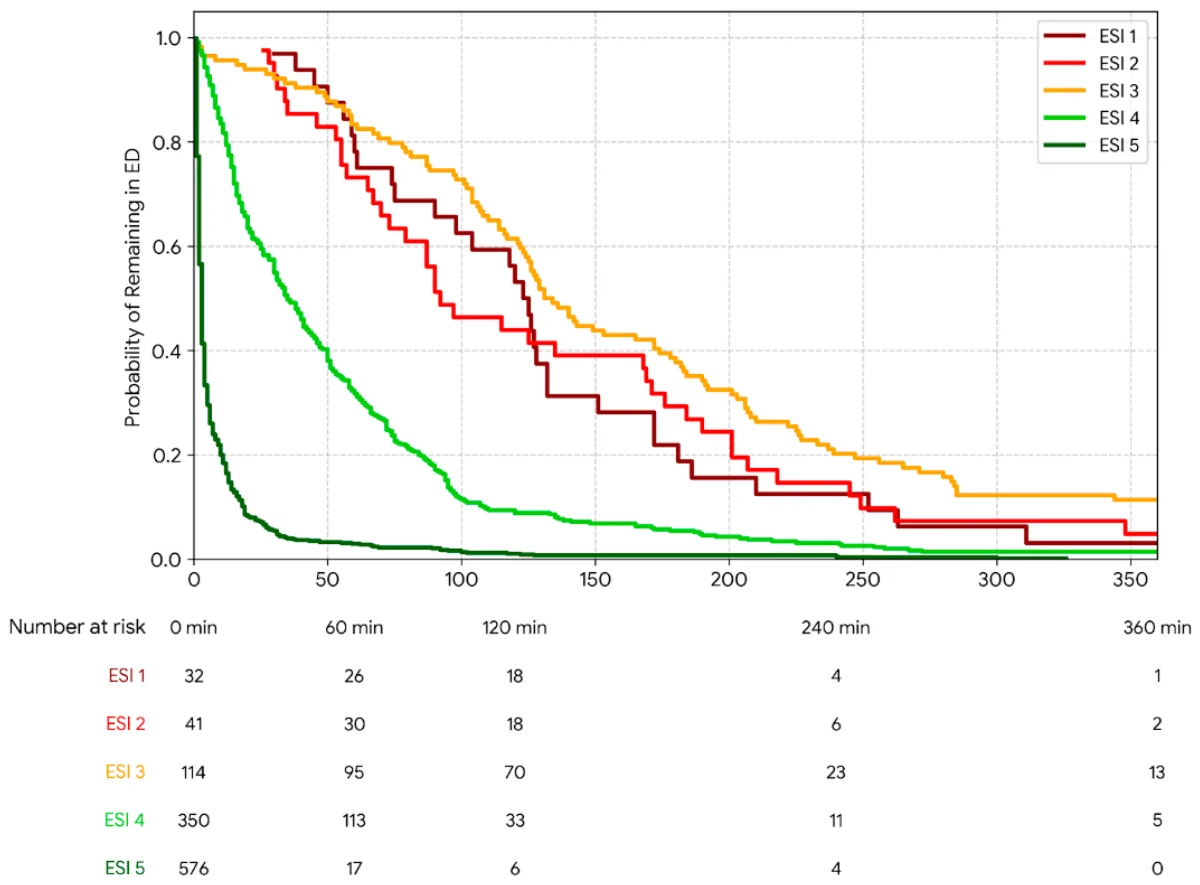
Kaplan–Meier survival curve for emergency department retention stratified by ESI triage zones. The event of interest was discharge from the ED. Red zone (ESI 1–2) represents the highest acuity; Yellow zone (ESI 3) represents urgent cases; and Green zone (ESI 4–5) represents the lowest acuity. A statistically significant difference was observed across the triage zones (Log-rank *p* < 0.001). The plot demonstrates that Green zone patients are discharged rapidly within the first 60 min, whereas Yellow and Red zone patients exhibit substantially prolonged ED retention, with a notable proportion remaining beyond the 120 min observation window.

To visually demonstrate the impact of clinical acuity on ED-LOS, a Kaplan–Meier survival analysis was conducted comparing the three triage zones (Red, Yellow, and Green) based on the ESI classification. The event of interest was defined as discharge from the ED. The analysis revealed a statistically significant difference in time-to-discharge across the triage zones (Log-rank *p* < 0.001) (Figure 2).

As illustrated in Figure 2, the Green zone patients (ESI 4–5) exhibited a rapid decline in the proportion of patients remaining in the ED, with approximately 85% of patients discharged within the first 60 min. In contrast, the Yellow zone (ESI 3) and Red zone (ESI 1–2) patients demonstrated markedly longer ED-LOS, with their survival curves shifted substantially to the right. Notably, more than 60% of Yellow zone patients remained in the ED at the 120 min mark, indicating ongoing diagnostic workup, consultation, or admission processes. The number-at-risk table further corroborated these findings, with only a small fraction of high-acuity patients remaining after 240 min.

### 3.5. ROC Cut-Off Determination for ED-LOS

**Figure 3:**
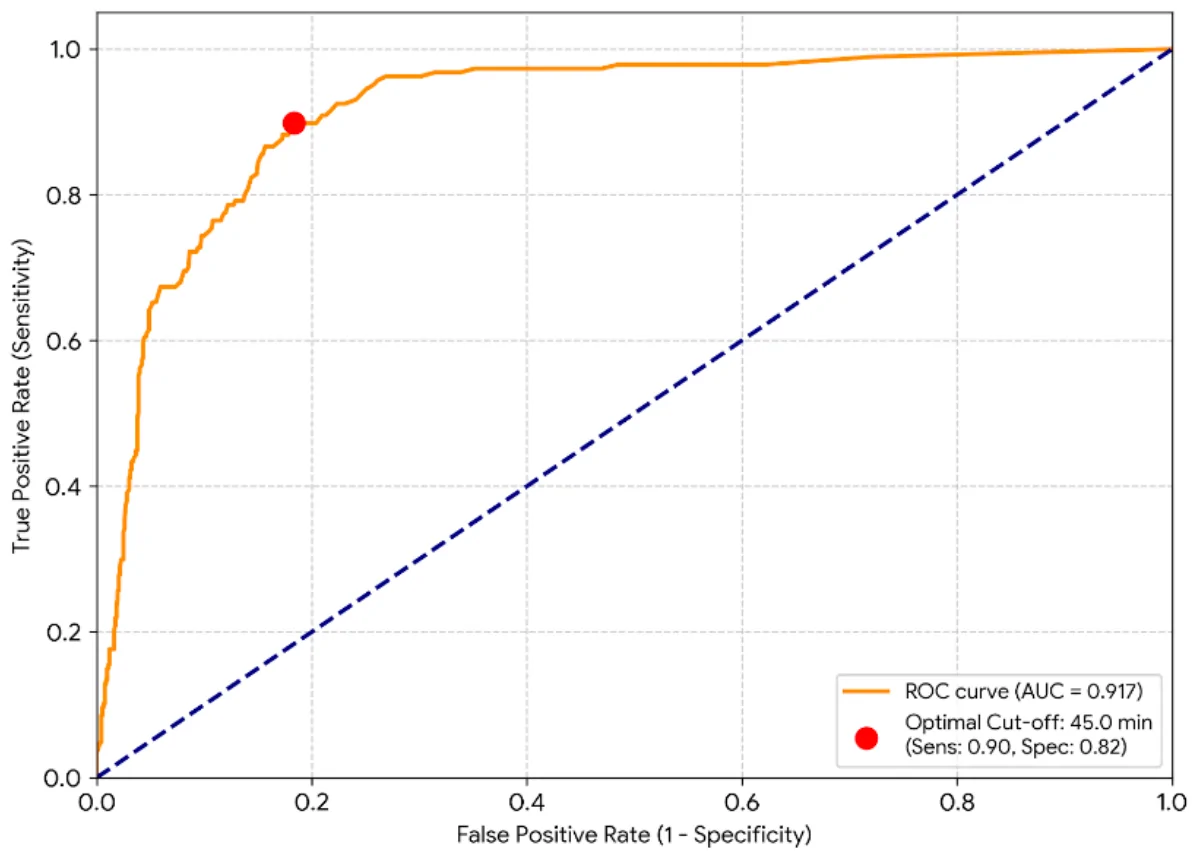
ROC curve for determination of emergency department length of stay threshold. The ROC curve evaluates ED length of stay as an indicator of high clinical acuity (ESI 1–3). The analysis demonstrates an excellent discriminative ability (AUC = 0.917), with the optimal Youden index cut-off identified at 45.0 min (Sensitivity: 0.90, Specificity: 0.82). The dotted navy line represents the line of no discrimination. Abbreviations: ESI: Emergency Severity Index; ROC: Reciever Operating Characteristic.

ROC curve analysis was performed to establish a data-driven threshold for ED-LOS, using high clinical acuity (ESI 1–3) as the reference standard. The analysis revealed that ED-LOS is an excellent discriminator for high-acuity cases, with an AUC of 0.917 (Figure 3). The Youden Index identified 45.0 min as the optimal cut-off, yielding a sensitivity of 89.8% and a specificity of 81.6%. Consequently, this ≥45 min threshold was utilized to dichotomize the outcome specifically for the binary predictive models.

### 3.6. Predictors of Prolonged ED Length of Stay

Univariate and multivariable Firth-penalized logistic regression analyses were performed to evaluate factors associated with an ED-LOS ≥ 45 min (Table 4). In the univariate analysis, advancing age (OR: 0.90, *p* < 0.001) and higher ESI scores (OR: 0.16, *p* < 0.001) were significantly inversely associated with prolonged stay. Among forensic types, out-of-vehicle accidents (OR: 26.43), intoxication (OR: 18.57), falls (OR: 15.14), in-vehicle accidents (OR: 14.48), suicide attempts (OR: 8.46), burns (OR: 8.47), sharp object injuries (OR: 6.30), and occupational accidents (OR: 4.78) all demonstrated significantly higher odds (all *p* < 0.001). Ambulance arrival (OR: 12.35, *p* < 0.001), autumn season (OR: 1.71, *p* = 0.003), and evening admission (15:00–20:59; OR: 1.40, *p* = 0.027) were also significant univariate predictors. Sex, sexual abuse, and assault did not reach statistical significance in the univariate analysis.

After adjusting for potential confounders in the multivariable model (Table 4), higher ESI score (aOR: 0.25, *p* < 0.001) and advancing age (aOR: 0.95, *p* = 0.008) remained independent protective factors. Significant independent risk factors included out-of-vehicle accidents (aOR: 8.82, *p* < 0.001), intoxication (aOR: 8.63, *p* = 0.002), burns (aOR: 7.53, *p* = 0.006), in-vehicle accidents (aOR: 5.57, *p* < 0.001), falls (aOR: 4.45, *p* = 0.003), occupational accidents (aOR: 4.27, *p* = 0.004), sharp object injuries (aOR: 2.38, *p* = 0.047), and assault (aOR: 1.96, *p* = 0.011). Ambulance arrival independently increased the odds of prolonged stay (aOR: 2.06, *p* = 0.004), while parent-accompanied presentation independently reduced the odds (aOR: 0.42, *p* = 0.006). Notably, the significant seasonal and admission time effects observed in the univariate analysis were no longer significant after adjustment, suggesting that these associations were explained by other clinical and operational factors.

### 3.7. Model Discrimination, Calibration, and Internal Validation

The multivariable Firth regression model demonstrated excellent discriminative ability for predicting ED-LOS ≥ 45 min, with an AUC of 0.936 (Figure S1). Using the Youden Index, the optimal probability threshold was 0.192, yielding a sensitivity of 94.1%, specificity of 79.7%, PPV of 66.9%, and NPV of 96.9%, with an overall accuracy of 84.1%.

**Figure 4:**
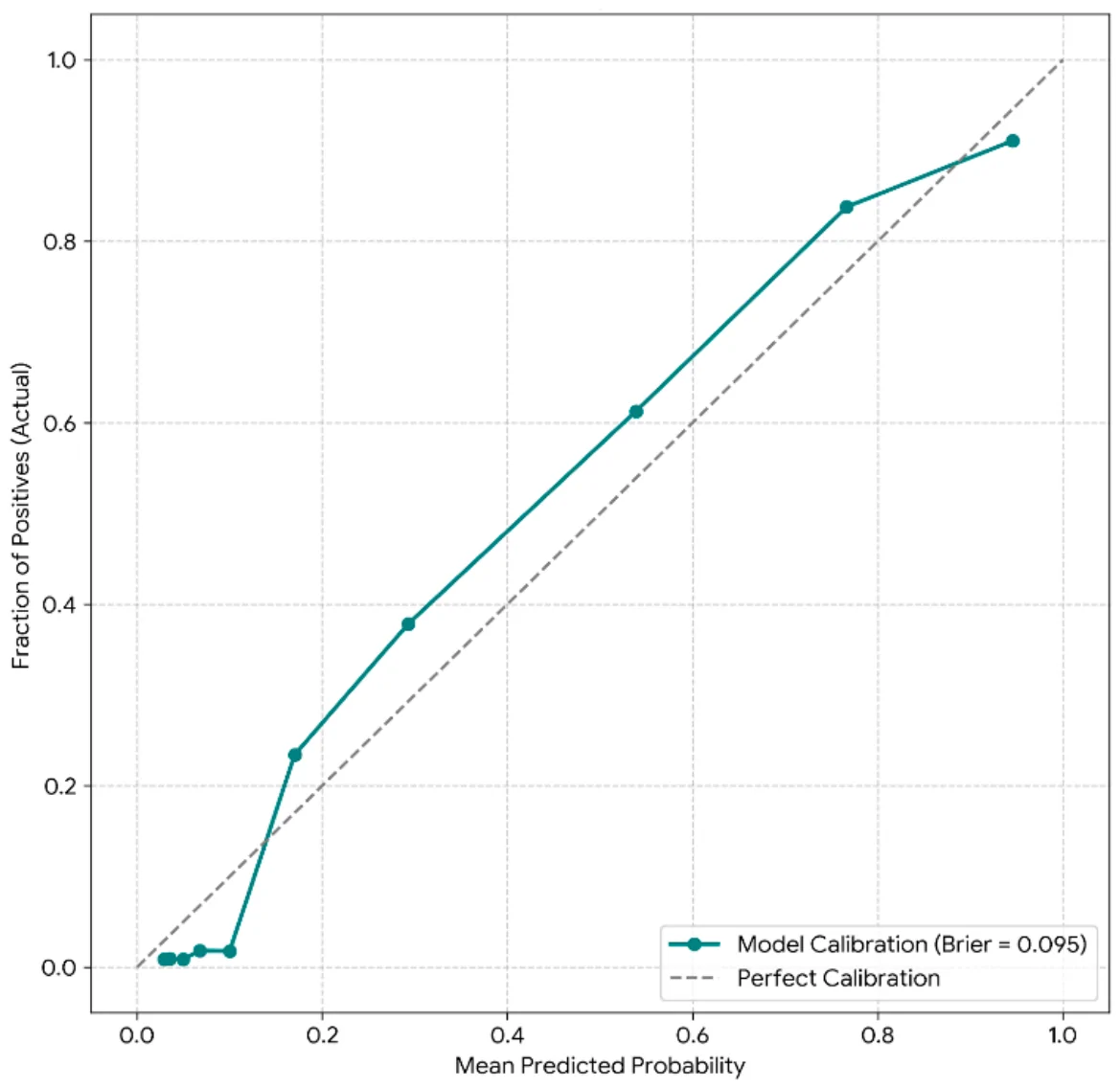
Calibration plot for the penalized regression model. The teal line indicates the model’s calibration against the dashed grey line (perfect calibration), demonstrating strong agreement between predicted and observed probabilities.

The model was calibrated with a Brier score of 0.095. Collinearity diagnostics confirmed the absence of multicollinearity (all VIF values < 2.5). The model explained a substantial proportion of variance (McFadden R^2^ = 0.496; Nagelkerke R^2^ = 0.645). Following 500-repetition bootstrap internal validation, the optimism-corrected AUC remained high at 0.929, with a corrected Brier score of 0.100, indicating excellent stability and generalizability without overfitting (Table S3). The calibration plot demonstrated strong agreement between the predicted and observed probabilities, with the model showing excellent calibration in high-risk cohorts (mean predicted probability > 0.3). Slight deviation was observed in the mid-range probabilities (Figure 4).

### 3.8. Clinical Utility and Decision Curve Analysis

**Figure 5:**
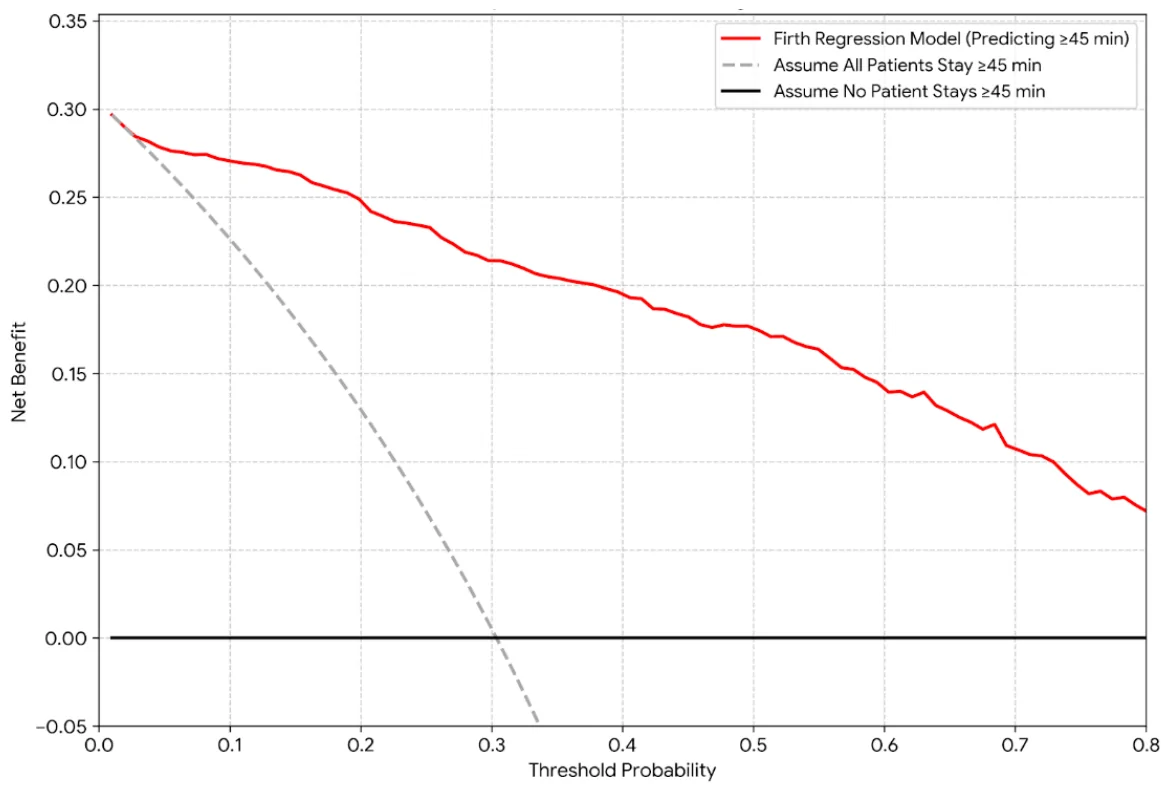
Decision curve analysis for predicting ED-LOS ≥ 45 min. The solid red line represents the net benefit of the Firth regression model, outperforming standard default strategies across clinically relevant thresholds.

DCA was employed to evaluate the practical clinical utility of the predictive model (Figure 5). The Firth regression model (red line) provided a consistently higher net clinical benefit compared to the “treat-all” and “treat-none” strategies across a wide range of threshold probabilities (0.01 to >0.80). This confirms that using the model to identify pediatric forensic cases likely to exceed the 45 min ED-LOS threshold enables EDs to optimize resource allocation without increasing false-positive burdens.

### 3.9. Machine Learning Predictive Modeling and Feature Importance

**Figure 6:**
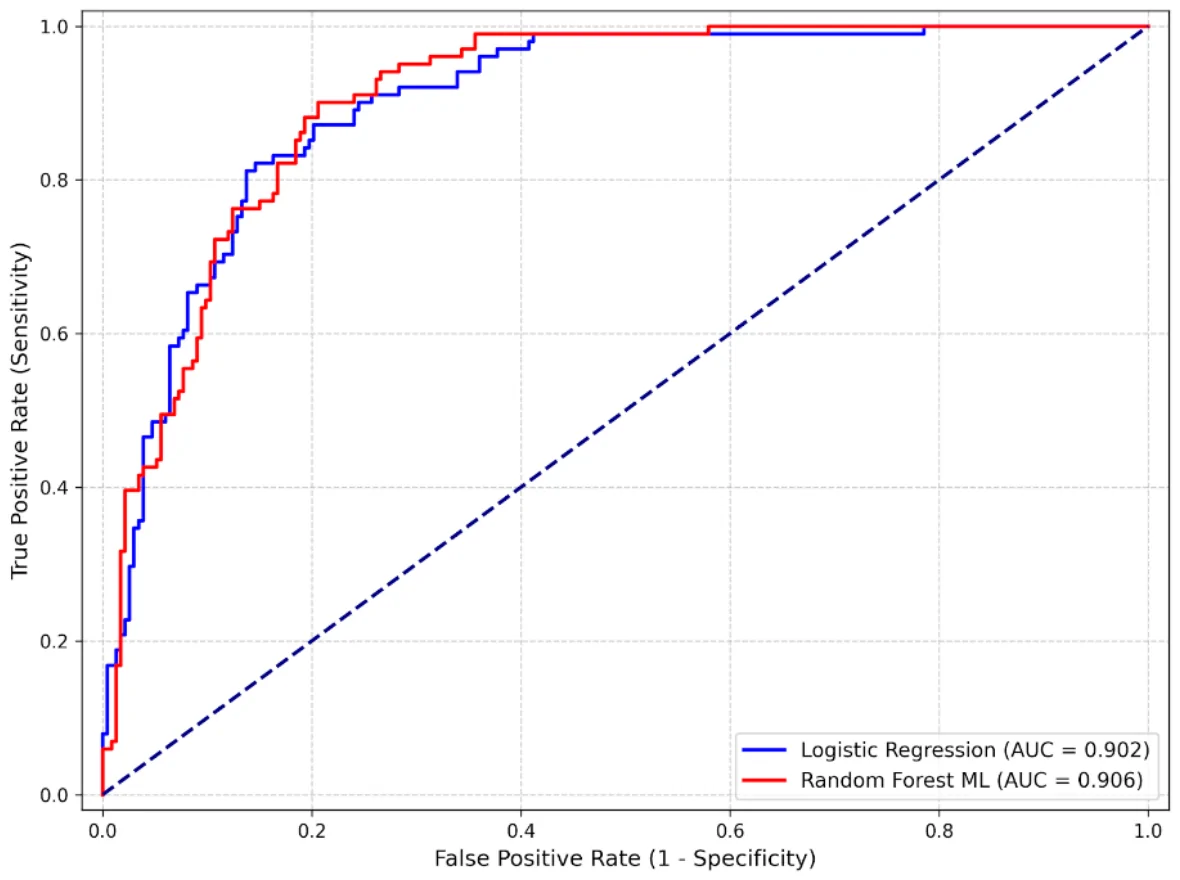
ROC comparison on the held-out test set: traditional regression vs. machine learning. The RF model (red line, AUC = 0.906) demonstrates robust, generalizable predictive accuracy comparable to the traditional regression model (blue line, AUC = 0.902) when evaluated on previously unseen data. Both models were trained on the same 70% training set and tested on the same 30% held-out test set (n = 334). The dotted navy line represents the line of no discrimination. The DeLong test confirmed no statistically significant difference between the two AUCs (*p* = 0.465). Abbreviations: ML: Model.

The RF classifier was developed and strictly evaluated on a held-out test set to capture complex, non-linear interactions without the risk of overfitting. Upon evaluation on the unseen test cohort, the RF algorithm demonstrated high discriminative ability, achieving an AUC of 0.906. This performance was competitive with the logistic regression model evaluated on the exact same test set (AUC = 0.902) (Figure 6). A DeLong test for correlated ROC curves confirmed that there was no statistically significant difference in discriminative performance between the RF algorithm and the logistic regression model (Z = 0.731, *p* = 0.465). This parity supports the structural reliability of the RF model on novel clinical data, ruling out the possibility of data leakage or overfitting.

**Figure 7:**
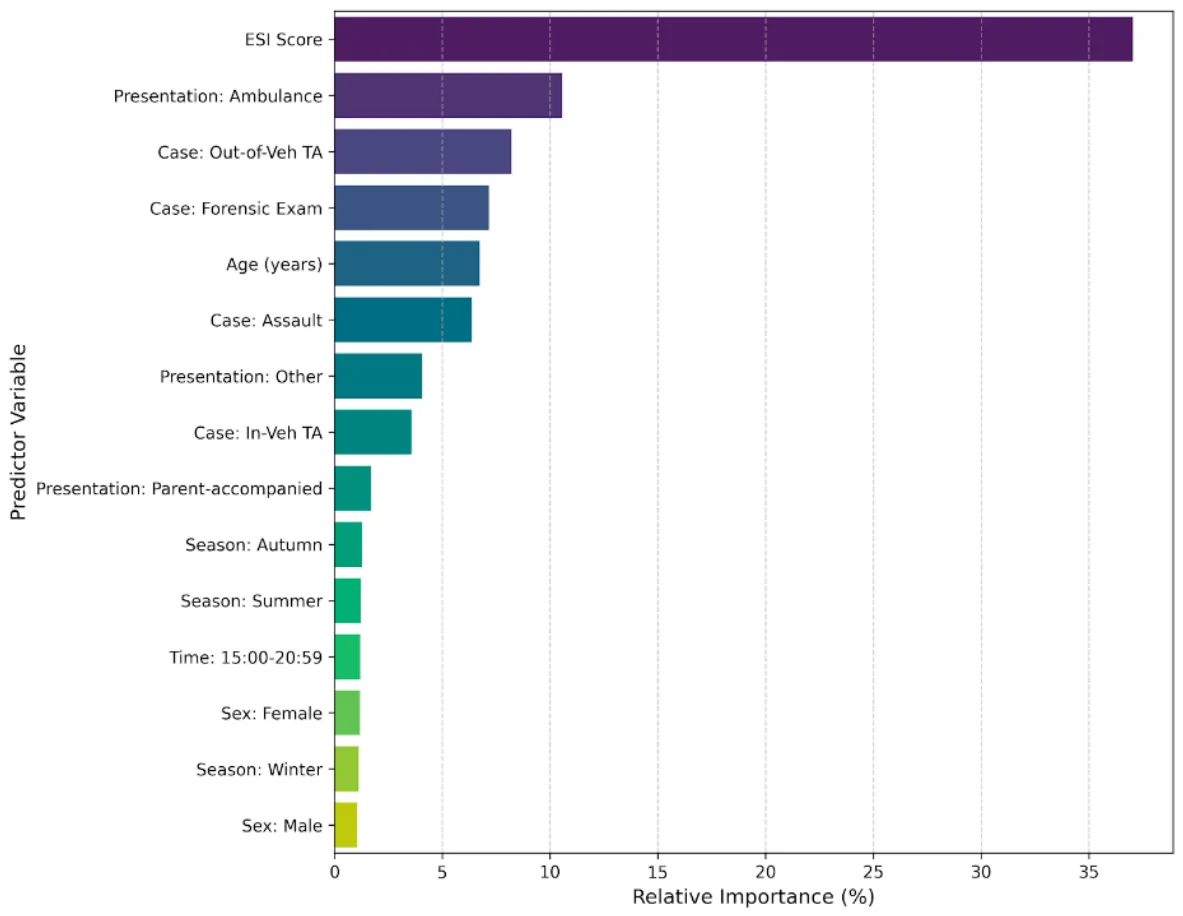
Random Forest feature importance for predicting ED-LOS. The bar chart highlights the relative percentage weight of independent predictors driving the algorithm’s operational triage decisions, quantified by the mean decrease in Gini impurity. The ESI score emerged as the predominant determinant (37.0%), followed by mode of presentation (ambulance arrival, 10.6%), out-of-vehicle TA (8.2%), forensic examinations (7.2%), and age (6.7%). These non-parametric rankings validate the regression-derived adjusted odds ratios. Abbreviations: ESI: Emergency Severity Index; TA: Traffic Accident.

Because the machine learning algorithm achieved a performance equivalent to the baseline regression, its internal decision-making metrics can be interpreted with confidence. Feature importance analysis quantified the specific mathematical weight of each independent clinical variable in driving ED-LOS. The ESI score emerged as the predominant determinant, accounting for over one-third (37.0%) of the model’s total predictive weight. This was followed by the mode of presentation (ambulance arrival, 10.6%), specific trauma mechanisms including out-of-vehicle traffic accidents (8.2%) and standard forensic examinations (7.2%), and patient age (6.7%). These non-parametric algorithmic rankings provide confirmatory evidence of the core clinical predictors (Figure 7).

## 4. Discussion

In this study, we identified several key findings that extend beyond descriptive epidemiology into predictive operational management. To establish the operational impact of predictors without the bias of dichotomization, we primarily employed a continuous-time Cox proportional hazards regression, which confirmed that the ESI score (HR: 1.954), intoxication (HR: 0.126), burns (HR: 0.127), and out-of-vehicle traffic accidents (HR: 0.237) are determinants of discharge speed. To translate these continuous-time findings into an actionable triage tool, we applied ROC analysis to establish a 45 min operational threshold (AUC 0.917) and used Firth-penalized logistic regression. The same predictors, ESI score (aOR: 0.25), out-of-vehicle traffic accidents (aOR: 8.82), intoxication (aOR: 8.63), burns (aOR: 7.53), and ambulance arrival (aOR: 2.06), emerged as independent predictors of ED-LOS ≥ 45 min, while parent-accompanied presentation (aOR: 0.42) was protective. This was further supported by the RF model, which confirmed ESI as the dominant predictor (37.0% feature importance) and demonstrated an AUC of 0.906 on the held-out test data, comparable to logistic regression (AUC 0.902, *p* = 0.465).

Consistent with the literature, our cohort demonstrated male predominance (65.3%), aligning with the 53.4–75.9% range reported across multiple studies [1,6,7,17,18,19,20,21,22,23,24]. This male excess likely reflects a combination of biological factors (greater physical activity, higher risk-taking behaviors) and sociocultural influences, including greater autonomy afforded to male children and increased exposure to trauma mechanisms such as interpersonal violence and traffic accidents [23,24,25]. Notably, studies with toxicological predominance reported balanced or female-majority distributions [2,6,7], underscoring the role of institutional case-mix in shaping demographic profiles.

Adolescence constituted the highest-risk developmental period in our cohort (71.5%, median age 15 years), consistent with national and international data [18,23,24,26,27]. Assault was the most common forensic presentation overall, accounting for 36.7% (n = 409) of cases, with the majority occurring in the adolescent age group (73.1%). These assault cases represented a heterogeneous mix of socio-medical and forensic dynamics, including peer-to-peer conflicts in school and social settings, large-scale physical altercations, assaults associated with robbery or extortion, and domestic violence cases. The assault rate observed in our study (36.7%) is higher than that reported in some European cohorts (typically 15–25%) [1,17], while being comparable to other studies from Turkey [19,21,23]. This variation likely reflects geographic, sociodemographic, and institutional differences in pediatric forensic epidemiology, as well as potential differences in case categorization and reporting practices across healthcare systems. The predominance of assault in our adolescent population is consistent with WHO data emphasizing interpersonal violence as a leading injury mechanism in adolescents, particularly in low- and middle-income countries [26,27]. However, age-specific etiological profiles diverged from certain descriptive studies. While falls are typically reported as the predominant forensic mechanism in children aged 0–5 years, our cohort demonstrated traffic accidents as the most frequent cause in this age group [1,28]. This discrepancy may reflect institutional variation, potential under-reporting of pediatric falls as forensic events by healthcare personnel, and possible familial concealment of domestic injuries.

Comparisons with the specific regional literature further illustrate how the institutional case-mix shapes these findings. For instance, Esen and Doğan’s non-trauma cohort was dominated by poisonings (90.8%), featuring a balanced sex distribution, a younger demographic, a spring peak, and higher mortality (1.34% vs. our 0.7%) [18]. Conversely, Demirel and Akpınar’s blunt trauma cohort captured higher kinetic-energy mechanisms, primarily falls, resulting in a 4.4% mortality rate [20]. Together, these comparisons reflect variations in pediatric forensic profiles, highlighting the necessity of transition from descriptive profiling to actionable predictive modeling.

The burden imposed by pediatric forensic cases on EDs extends beyond acute medical intervention. The socio-medical context of this burden is shaped by a broad network of factors, including family dynamics, gender roles, economic inequalities, and cultural norms [29,30]. Globally, United Nations Children’s Fund report that 1.6 billion children are regularly subjected to physical or psychological discipline at home, with an estimated 130,000 deaths from violence annually [31]. Peer bullying and cyberbullying represent additional dimensions, affecting 11–15% of children in some regions [32]. Socioeconomic vulnerability further compounds these risks; patients from low socioeconomic backgrounds constitute 70.1% of forensic cases and face a 2.5-fold higher likelihood of severe injury [29]. National Child Abuse and Neglect Data System data confirm caregiver substance use (25.1%) and domestic violence (27.6%) as leading risk factors for severe child abuse [33], while children in disadvantaged neighborhoods have 1.31-fold higher rates of confirmed physical abuse [34]. In operational terms, this burden in our setting encompasses administrative documentation requiring meticulous medico-legal forms; multidisciplinary coordination with social workers, psychiatrists, and law enforcement; prolonged pediatric patient retention until safety can be guaranteed; and reduced ED throughput and efficiency. However, we acknowledge that this burden varies across healthcare systems. Trauma-predominant cohorts, such as ours, differ from toxicology-predominant cohorts, and the magnitude of this burden depends on the institutional case-mix, local medicolegal requirements, and sociodemographic factors. This variability supports developing institution-specific predictive models rather than applying universal thresholds.

The overall median ED-LOS in our cohort was short at 12 min (IQR: 3–60.5), reflecting that 83.2% of patients were triaged to the Green zone (ESI 4–5) and required minimal diagnostic or therapeutic intervention. This operational efficiency is consistent with the predominance of low-acuity forensic examinations (26.7%) and minor assault-related injuries, which constitute the majority of our cohort. Hospitalization was required in 9.3% of cases, with an overall mortality rate of 0.7% (n = 8). These rates are higher than those reported for the secondary care cohort (hospitalization: 1.2–3.2%; mortality: 0.1%) [8,17,19,20]. However, they are markedly lower than those reported for toxicological and pediatric intensive care cohorts (hospitalization: 26.9–33.1%; mortality: 0.6–3.9%) [6,7], supporting the distinct operational dynamics of trauma-focused vs. non-trauma forensic populations.

Temporal analysis revealed a consistent evening peak in admissions (15:00–03:00), mirroring prior studies [7,35]. This pattern is likely multifactorial, reflecting increased ED utilization during evening hours, reduced outdoor activity during late-night hours, parental occupational schedules, and the perception of emergency services as more accessible for medico-legal documentation. Seasonal variation demonstrated a summer peak (34.0%), consistent with the trauma-associated seasonal patterns reported in other studies [7,17,19,20,35], while a study with toxicological predominance consistently reported spring peaks [18]. Notably, in the multivariable model, seasonal effects lost statistical significance, suggesting that they are largely mediated by the case-mix composition (e.g., higher rates of outdoor trauma-related injuries during summer months).

Cox regression identified the ESI score as the predominant determinant of discharge speed, supporting its established relationship with resource utilization, hospitalization rates, and patient flow [36,37]. Specifically, higher ESI scores (indicating lower clinical acuity) were associated with a significantly increased hazard of discharge (HR: 1.954), demonstrating that patients with lower acuity are discharged nearly twice as fast for each 1-point increase in ESI score.

Consistent with the Cox findings, our binary Firth model identified the ESI-3 category as the group exhibiting the longest median ED-LOS (132.5 min), consistent with large-scale studies by Sax et al. [15] and Frankenberger et al. [38], which highlight ESI-3’s diagnostic ambiguity, high rates of overtriage, and consequent resource overconsumption. In contrast, Green zone patients (ESI-4 and ESI-5) exhibited a substantially shorter ED-LOS (median 7 min), reflecting the limited diagnostic and therapeutic interventions required for low-acuity presentations. This gradient in ED-LOS across acuity levels is concordant with the broader literature, which demonstrates that lower ESI scores correspond to increased diagnostic workup, consultation needs, and treatment intensity [36,37].

Specific trauma mechanisms emerged as independent drivers of prolonged discharge in both the Cox and Firth models. The most pronounced delays were observed for intoxication (HR: 0.126), burns (HR: 0.127), and out-of-vehicle traffic accidents (HR: 0.237), indicating that these patients are discharged approximately 87% slower than standard forensic examination cases. The consistency between these continuous-time HRs and the binary Firth-adjusted ORs (intoxication aOR: 8.63; burns aOR: 7.53; out-of-vehicle TA aOR: 8.82) provides implications that these mechanisms delay discharge at every time point.

These findings align with international cohorts. Gibbs et al. [39] similarly identified burns and corrosive injuries as primary predictors of prolonged hospitalizations in pediatric trauma, while Kumar et al. [40] demonstrated that pediatric burns require time-intensive procedural interventions prior to disposition. The operational burden associated with intoxication reflects the need for toxicological screening, decontamination, and observation, consistent with the longer ED-LOS observed in this subgroup. Out-of-vehicle TA typically requires comprehensive imaging and surgical consultation, which contribute to increased ED-LOS.

Furthermore, our models identified younger patient age and higher clinical acuity as independent risk factors for ED-LOS ≥ 45 min, a finding that may be partly explained by the inherent challenges of evaluating infants and young children. In this age group, history taking is often difficult, physical examination requires greater time and attention, and suspected cases of abuse or neglect necessitate multidisciplinary involvement, all of which can prolong ED assessment [41].

Ambulance arrival increased the odds of ED-LOS ≥ 45 min (aOR: 2.06), consistent with findings from Sax et al. [15] and Pepele et al. [42], who demonstrated that EMS-transported pediatric trauma patients are paradoxically at higher risk for undertriage and often require resource-intensive diagnostic evaluations. Notably, however, in the continuous-time Cox model, ambulance arrival was not significantly associated with the overall discharge trajectory (HR: 0.879, *p* = 0.176). This suggests that, while ambulance cases are more likely to exceed the 45 min threshold, the overall time-to-discharge is not independently slower once the specific trauma mechanism is accounted for. This finding suggests that ambulance arrival serves as a marker for injury severity rather than an independent driver of prolonged stay.

Conversely, parent-accompanied presentation independently reduced the odds of ED-LOS ≥ 45 min (aOR: 0.42), as corroborated by our Cox regression (HR: 1.303, *p* = 0.025). This protective effect likely reflects enhanced history-taking efficiency, improved child cooperation, and more rapid decision-making when caregivers are present. This finding has practical implications for ED triage protocols, suggesting that encouraging caregiver presence may facilitate throughput.

Our model also showed clinical utility, with an NPV of 96.9% and favorable net benefit across DCA. While large-scale trauma registries have employed machine learning to predict multi-day inpatient hospitalizations [39], our study adapts ensemble algorithms to the ED micro-operational level, targeting rapid throughput requirements in acute forensic and trauma settings. The recent literature has highlighted the low baseline accuracy of human-assigned ESI and proposed artificial intelligence as a corrective tool at triage [15,38,43]. Our approach differs in that we translate real-world ESI assignments into predictors of ED-LOS, rather than attempting to improve triage accuracy itself. This may offer a complementary strategy for ED workflow optimization.

### Limitations

This study has several limitations. First, the single-center, retrospective design limits the generalizability of our specific 45 min threshold to institutions with different patient volumes, staffing levels, or operational workflows. Second, the retrospective nature restricts our findings to associations rather than causal relationships and precludes adjustment for unmeasured institutional factors such as bed availability or radiology turnaround times. Third, certain forensic categories (notably sexual abuse, intoxication, and burns) had small sample sizes. Therefore, despite the use of penalized regression, these specific estimates should be interpreted cautiously. Fourth, our analysis focused exclusively on operational throughput and lacked data on post-discharge, patient-centered outcomes. Finally, deriving the 45 min threshold from ESI scores inherently introduces mathematical coupling in our binary predictive models. However, we mitigated this bias by utilizing a continuous-time Cox regression, which analyzes exact time-to-discharge without dichotomization and independently confirmed the same predictive trends.

## 5. Conclusions

This study proposes a framework for predicting increased ED-LOS in pediatric forensic cases. In our study, Cox regression offered evidence that ESI score and specific trauma mechanisms independently modulated discharge speed, suggesting that these associations reflect operational patterns. ESI was the dominant predictor, with burns, intoxication, and out-of-vehicle accidents as key risk factors, and parent-accompanied presentation as protective. DCA benefits suggest that it may support efficient resource allocation and early identification of high-risk cases. These findings may provide a foundation for triage stratification, resource allocation, and forensic process optimization, with potential implications for health policy and emergency preparedness.

## Figures and Tables

**Table 1:** Baseline demographic and clinical characteristics of pediatric forensic cases.

**Demographics**		
Number of cases		1113 (100%)
Age, years		15 (10–16)
Gender	Male	727 (65.3%)
	Female	386 (34.7%)
Developmental Period	Infancy	50 (4.5%)
	Early Childhood	106 (9.5%)
	Middle Childhood	161 (14.5%)
	Adolescence	796 (71.5%)
**Clinical Characteristics**		
ESI score		5 (4–5)
Triage Area	Red zone	73 (6.6%)
	Yellow zone	114 (10.2%)
	Green zone	926 (83.2%)
Treatment Type	Outpatient management	1010 (90.7%)
	Hospitalization	103 (9.3%)
**Admission Characteristics**		
Season	Spring	317 (28.5%)
	Summer	378 (34.0%)
	Autumn	228 (20.5%)
	Winter	190 (17.1%)
Day of Admission	Weekday	840 (75.5%)
	Weekend	273 (24.5%)
Time of Admission	03:00–08:59	70 (6.3%)
	09:00–14:59	346 (31.1%)
	15:00–20:59	434 (39.0%)
	21:00–02:59	263 (23.6%)
Mode of Presentation	Ambulance (EMS)	166 (14.8%)
	Self-presentation	52 (4.7%)
	Parent accompanied	93 (8.4%)
	Other	802 (72.1%)
**Outcomes**		
Length of stay, min		12 (3–60.5)
Length of Stay Categories (5-group)	<10 min	525 (47.2%)
	10–30 min	192 (17.3%)
	30–60 min	119 (10.7%)
	60–120 min	130 (11.7%)
	>120 min	147 (13.2%)
Mortality	Emergency Department	3 (37.5%)
	Inpatient Wards/ICU	5 (62.5%)
Referral Status	No referral	1084 (97.4%)
	Referred by ambulance	27 (2.4%)
	Self-transfer	2 (0.2%)

Data are presented as medians (IQR) for continuous variables and as frequencies n (%) for categorical variables. Abbreviations: ESI: Emergency Severity Index; EMS: emergency medical services; ICU: Intensive Care Unit; IQR: interquartile range.

**Table 2:** Distribution of forensic case types across developmental periods.

Forensic Case Type	Infancy (n = 50)	Early Childhood (n = 106)	Middle Childhood (n = 161)	Adolescence (n = 796)	Total (N = 1113)	*p*-Value
**Assault**	12 (24.0)	30 (28.3)	68 (42.2)	299 (37.6)	409 (36.7)	**0.028**
**Forensic Examination**	4 (8.0)	7 (6.6)	14 (8.7)	272 (34.2)	297 (26.7)	**<0.001**
**Out-of-Vehicle Traffic Accident**	6 (12.0)	26 (24.5)	37 (23.0)	82 (10.3)	151 (13.6)	**<0.001**
**In-Vehicle Traffic Accident**	12 (24.0)	21 (19.8)	32 (19.9)	70 (8.8)	135 (12.1)	**<0.001**
**Sharp Object Injury**	5 (10.0)	8 (7.5)	4 (2.5)	27 (3.4)	44 (4.0)	**0.019**
**Falls**	7 (14.0)	7 (6.6)	3 (1.9)	9 (1.1)	26 (2.3)	**<0.001**
**Occupational Accident**	0 (0.0)	0 (0.0)	1 (0.6)	23 (2.9)	24 (2.2)	0.064
**Intoxication**	3 (6.0)	4 (3.8)	0 (0.0)	2 (0.3)	9 (0.8)	**<0.001**
**Suicide Attempt**	0 (0.0)	0 (0.0)	1 (0.6)	7 (0.9)	8 (0.7)	0.696
**Burns**	0 (0.0)	2 (1.9)	0 (0.0)	4 (0.5)	6 (0.5)	0.191
**Sexual Abuse**	1 (2.0)	1 (0.9)	1 (0.6)	1 (0.1)	4 (0.4)	0.098

Data are presented as n (column percentage), representing the proportion of each forensic case type distributed across age groups. *p*-values were derived from Pearson Chi-Square tests (df = 3 for all comparisons). Statistically significant values (*p* < 0.05) are indicated in bold.

**Table 3:** Cox Proportional Hazards Regression Analysis for ED-LOS as a Continuous Time Variable.

Independent Predictors	Hazard Ratio (HR)	95% CI	*p*-Value
Age (continuous)	1.022	1.007–1.037	**0.004**
ESI Score (continuous)	1.954	1.771–2.155	**<0.001**
Forensic Case Type (Ref: Forensic Examination)			
Out-of-Vehicle Traffic Accident	0.237	0.185–0.304	**<0.001**
In-Vehicle Traffic Accident	0.283	0.221–0.363	**<0.001**
Sexual Abuse	0.262	0.097–0.708	**0.008**
Assault	0.511	0.432–0.605	**<0.001**
Falls	0.301	0.189–0.477	**<0.001**
Intoxication	0.126	0.062–0.255	**<0.001**
Occupational Accident	0.346	0.218–0.550	**<0.001**
Sharp Object Injury	0.478	0.339–0.675	**<0.001**
Suicide Attempt	0.509	0.244–1.060	0.071
Burns	0.127	0.056–0.288	**<0.001**
Mode of Presentation (Ref: Other)			
Ambulance (EMS)	0.879	0.729–1.059	0.176
Self-presentation	1.004	0.734–1.375	0.979
Parent-accompanied	1.303	1.034–1.643	**0.025**

HR < 1 indicates slower discharge; HR > 1 indicates faster discharge. Bold *p*-values indicate statistical significance (*p* < 0.05). All patients were uncensored as they completed their ED episode. Reference categories are indicated in parentheses. Abbreviations: CI: confidence interval; ESI: Emergency Severity Index; EMS: emergency medical services; Ref: reference category.

**Table 4:** Univariate and Multivariable Firth-Penalized Logistic Regression for Predictors of ED-LOS ≥ 45 min.

Variable	Category	Univariate		Multivariable	
		OR (95% CI)	*p*-Value	aOR (95% CI)	*p*-Value
Age (years)	-	0.90 (0.88–0.92)	**<0.001**	0.95 (0.91–0.99)	**0.008**
ESI Score (1–5)	-	0.16 (0.13–0.20)	**<0.001**	0.25 (0.19–0.33)	**<0.001**
Gender	Female (Ref)	1.00	-	-	-
	Male	0.91 (0.70–1.18)	0.471	—	—
Forensic Case Type	Forensic Examination (Ref)	1.00	-	1.00	-
	Out-of-Vehicle Traffic Accident	26.43 (16.31–42.84)	**<0.001**	8.82 (4.77–16.31)	**<0.001**
	In-Vehicle Traffic Accident	14.48 (9.05–23.16)	**<0.001**	5.57 (3.03–10.23)	**<0.001**
	Sexual Abuse	2.76 (0.62–12.40)	0.184	2.75 (0.57–13.20)	0.205
	Assault	1.47 (0.96–2.26)	0.076	1.96 (1.17–3.31)	**0.011**
	Falls	15.14 (6.87–33.37)	**<0.001**	4.45 (1.65–12.05)	**0.003**
	Intoxication	18.57 (5.84–59.06)	**<0.001**	8.63 (2.17–34.34)	**0.002**
	Occupational Accident	4.78 (2.10–10.88)	**<0.001**	4.27 (1.60–11.41)	**0.004**
	Sharp Object Injury	6.30 (3.29–12.07)	**<0.001**	2.38 (1.01–5.60)	**0.047**
	Suicide Attempt	8.46 (2.67–26.80)	**<0.001**	1.19 (0.27–5.32)	0.818
	Burns	8.47 (2.41–29.73)	**<0.001**	7.53 (1.78–31.85)	**0.006**
Season	Spring (Ref)	1.00	-	1.00	-
	Summer	1.09 (0.79–1.51)	0.592	1.19 (0.76–1.85)	0.440
	Autumn	1.71 (1.20–2.44)	**0.003**	1.09 (0.65–1.85)	0.735
	Winter	1.12 (0.76–1.66)	0.557	1.23 (0.71–2.12)	0.466
Mode of Presentation	Other (Ref)	1.00	-	1.00	-
	Ambulance (EMS)	12.35 (8.34–18.27)	**<0.001**	2.06 (1.25–3.39)	**0.004**
	Self-presentation	1.18 (0.64–2.19)	0.598	0.73 (0.33–1.64)	0.448
	Parent accompanied	1.24 (0.77–2.00)	0.382	0.42 (0.23–0.79)	**0.006**
Admission Time	09:00–14:59 (Ref)	1.00	-	1.00	-
	03:00–08:59	0.85 (0.48–1.48)	0.556	0.92 (0.40–2.14)	0.848
	15:00–20:59	1.40 (1.04–1.88)	**0.027**	1.11 (0.73–1.68)	0.626
	21:00–02:59	0.74 (0.52–1.06)	0.100	0.88 (0.53–1.45)	0.604

Bold *p*-values indicate statistical significance (*p* < 0.05). The multivariable model was adjusted for all other variables shown. Abbreviations: aOR: adjusted odds ratio; OR: odds ratio; CI: confidence interval; ED: emergency department; ESI: Emergency Severity Index; EMS: emergency medical services; Ref: reference category.

## Data Availability

The raw data supporting the conclusions of this article will be made available by the authors upon request.

## Supplementary Materials

The following supporting information can be downloaded at https://www.mdpi.com/article/10.3390/jcm15187110/s1: Figure S1: ROC Curve of the Firth Penalized Multivariable Model; Table S1: Relationship Between ESI Score and Emergency Department Length of Stay; Table S2: Comparison of Emergency Department Length of Stay Across Triage Areas; Table S3: Model Goodness-of-Fit and Internal Validation.

## Abbreviations

The following abbreviations are used in this manuscript:

ED	Emergency Department
ED-LOS	Emergency Department Length of Stay
EMS	Emergency Medical Services
ESI	Emergency Severity Index
ICU	Intensive Care Unit
STROBE	Strengthening the Reporting of Observational Studies in Epidemiology
TRIPOD	Transparent Reporting of a Multivariable Prediction Model for Individual Prognosis or Diagnosis
WHO	World Health Organization

## References

[1] Taplak A.Ş., Tubaş F., Polat S. (2020). A Retrospective Records-Based Cohort of 1,082 Pediatric Forensic Cases Presenting to the Emergency Department. J. Emerg. Nurs..

[2] World Health Organization Global Status Report on Preventing Violence Against Children 2020. https://www.who.int/teams/social-determinants-of-health/violence-prevention/global-status-report-on-violence-against-children-2020.

[3] Hillis S., Mercy J., Amobi A., Kress H. (2016). Global Prevalence of Past-Year Violence Against Children: A Systematic Review and Minimum Estimates. Pediatrics.

[4] Turkish Statistical Institute Statistics on Children 2023. https://data.tuik.gov.tr/Bulten/Index?p=Istatistiklerle-Cocuk-2023-53679.

[5] United Nations Children’s Fund (2017). A Familiar Face: Violence in the Lives of Children and Adolescents.

[6] Arslan İ., Demir K.İ. (2022). Evaluation of Forensic Cases Presented to the Pediatric Emergency Department. Turk. J. Emerg. Med..

[7] Polat S., Terece C., Yaman A., Gürpınar K. (2021). Evaluation of Forensic Cases in the Pediatric Intensive Care Unit. Med. Bull. Sisli Etfal Hosp..

[8] Yılmaz G., Alemdar D.K. (2021). Evaluation of Pediatric Forensic Cases Admitted to the Emergency Department in Turkey: A Retrospective Analysis. J. Forensic Nurs..

[9] Gilboy N., Tanabe P., Travers D., Rosenau A.M. (2012). Emergency Severity Index (ESI): A Triage Tool for Emergency Department Care, Version 4. Implementation Handbook, 2012 Edition.

[10] von Elm E., Altman D.G., Egger M., Pocock S.J., Gøtzsche P.C., Vandenbroucke J.P. (2007). Strengthening the Reporting of Observational Studies in Epidemiology (STROBE) Statement: Guidelines for Reporting Observational Studies. BMJ.

[11] Collins G.S., Reitsma J.B., Altman D.G., Moons K.G.M. (2015). Transparent Reporting of a Multivariable Prediction Model for Individual Prognosis or Diagnosis (TRIPOD): The TRIPOD Statement. BMJ.

[12] Ersoy B., Balcı Y., Gök Y., Ünüvar Göçeoğlu Ü. (2020). The Evaluation of Pediatric Patients with Forensic Reports in a Forensic Medicine Out Patient Clinic. MMJ.

[13] Koç S., Can M. (2011). Forensic Medicine in Primary Care [Birinci Basamakta Adli Tıp].

[14] Uygun E., Çelik K., Elumar F., Kaya H. (2024). Evaluation of the Procedure and Content of Forensic Reports Issued for Judicial Cases in Emergency Departments. Med. Sci. Discov..

[15] Sax D.R., Warton E.M., Kene M.V., Ballard D.W., Vitale T.J., Timm J.A., Adams E.S., McGauhey K.R., Pines J.M., Reed M.E. (2024). Emergency Severity Index Version 4 and Triage of Pediatric Emergency Department Patients. JAMA Pediatr..

[16] Heinze G., Schemper M. (2002). A Solution to the Problem of Separation in Logistic Regression. Stat. Med..

[17] Alpaslan M., Baykan N. (2025). Analysis of Patients Evaluated Forensically in the Emergency Department and the Burden of Assault and Force Examinations on the Emergency Department. J. Med. Sci..

[18] Esen F.H., Doğan M. (2022). Forensic Cases in Pediatric Emergency Department: A Single Center Experience. J. Pediatr. Emerg. Intensive Care Med..

[19] Ökçesiz A., Kozacı N., Avcı M., Demirel B. (2018). The Evaluation of Pediatric Forensic Cases Presented to Emergency Department. Disaster Emerg. Med. J..

[20] Demirel M.E., Akpınar G. (2022). Characteristics of Pediatric Forensic Cases Caused by Blunt General Body Trauma Evaluated in the Emergency Department: A Single Center Experience. OTJHS.

[21] Bolat A. (2023). Evaluation of Forensic Cases Admitted to the Pediatric Emergency Department. J. Health Sci. Med..

[22] Zahavi A., Luckman J., Yassur I., Michowiz S., Goldenberg-Cohen N. (2016). Severe Cranial Neuropathies Caused by Falls from Heights in Children. Graefes Arch. Clin. Exp. Ophthalmol..

[23] Kadıoğlu E. (2018). Pediatric Forensic Cases: An Emergency Department Experience. Turk. J. Forensic Med..

[24] Kalkan E.A., Yıldırım A., Akdur O. (2016). Trauma and Intentional Injury Characteristics of Pediatric Forensic Cases Applying to Emergency Room. J. Clin. Anal. Med..

[25] Centers for Disease Control and Prevention Web-Based Injury Statistics Query and Reporting System (WISQARS). https://www.cdc.gov/injury/wisqars/index.html.

[26] World Health Organization (2015). The Global Strategy for Women’s, Children’s and Adolescents’ Health (2016–2030): Survive, Thrive, Transform.

[27] Li C., Jiao J., Hua G., Yundendorj G., Liu S., Yu H., Zhang L., Yang X., Liu L. (2024). Global Burden of All Cause-Specific Injuries Among Children and Adolescents from 1990 to 2019: A Prospective Cohort Study. Int. J. Surg..

[28] Centers for Disease Control and Prevention Data and Statistics (WISQARS): Injury Reports. https://wisqars.cdc.gov:8443/costT/.

[29] Çamcı M. (2026). Six-Year Analysis of Forensic Cases in the Emergency Department of a City Hospital: Demographic and Clinical Findings. OTJHS.

[30] Carannante A., Giustini M., Rota F., Bailo P., Piccinini A., Izzo G., Bollati V., Gaudi S. (2025). Intimate Partner Violence and Stress-Related Disorders: From Epigenomics to Resilience. Front. Glob. Womens Health.

[31] UNICEF FAST FACTS: Violence Against Children Widespread, Affecting Millions Globally. https://www.unicef.org/turkiye/en/press-releases/fast-facts-violence-against-children-widespread-affecting-millions-globally.

[32] UNICEF Two Thirds of Children Across Europe and Central Asia Experience Violent Discipline at Home, a Third Are Physically Punished. https://www.unicef.org/turkiye/en/press-releases/two-thirds-children-across-europe-and-central-asia-experience-violent-discipline.

[33] U.S. Department of Health and Human Services, Administration for Children and Families, Administration on Children, Youth and Families, Children’s Bureau Child Maltreatment 2024. https://acf.gov/cb/data-research/child-maltreatment.

[34] Hughes Garza H., Piper K.E., Lawson K.A. (2026). Physical Abuse of Young Children Reported by Medical Professionals in the United States 2014–2023. Front. Pediatr..

[35] Korkmaz T., Erkol Z., Kahramansoy N. (2014). Evaluation of Pediatric Forensic Cases in Emergency Department: A Retrospective Study. Med. Bull. Haseki.

[36] Travers D.A., Waller A.E., Katznelson J., Agans R. (2009). Reliability and Validity of the Emergency Severity Index for Pediatric Triage. Acad. Emerg. Med..

[37] Green N.A., Durani Y., Brecher D., DePiero A., Loiselle J., Attia M. (2012). Emergency Severity Index Version 4: A Valid and Reliable Tool in Pediatric Emergency Department Triage. Pediatr. Emerg. Care.

[38] Frankenberger W.D., Zorc J.J., Ten Have E.D., Brodecki D., Faig W.G. (2024). Triage Accuracy in Pediatrics Using the Emergency Severity Index. J. Emerg. Nurs..

[39] Gibbs D., Ehwerhemuepha L., Moreno T., Guner Y., Yu P., Schomberg J., Wallace E., Feaster W. (2021). Prolonged Hospital Length of Stay in Pediatric Trauma: A Model for Targeted Interventions. Pediatr. Res..

[40] Kumar N., Oag K., Vitale L., Malaniak M., Klein J., Ridelman E., Shanti C. (2025). Factors Influencing Emergency Department Length of Stay in Pediatric Burn Patients. J. Burn Care Res..

[41] Christian C.W., Committee on Child Abuse and Neglect, American Academy of Pediatrics (2015). The Evaluation of Suspected Child Physical Abuse. Pediatrics.

[42] Pepele M.S., Derya S., Murat M., Akdemir A., Yücel N. (2026). Pediatric Trauma in the Emergency Department: Clinical Risk Stratification, CT Utilization and Radiation Burden in a Tertiary Care Cohort. J. Clin. Med..

[43] Aygün E., İmdat A., Dalgıç N. (2026). Should We Leave Paediatric Emergency Triage to Artificial Intelligence? A Comparison of ChatGPT 4o and Grok 3. Front. Pediatr..

