# Supplementary material for: Evaluating Triage Acuity and Injury Mechanisms as Drivers of Emergency Department Length of Stay in Pediatric Forensic Cases
Source: J Clin Med. 2026 Sep 13;15(18):7110. doi: 10.3390/jcm15187110 (PMC13607831; doi:10.3390/jcm15187110)
Supplement: Supplementary file 1 [file jcm-15-07110-s001.zip › jcm-4545339-supplementary.pdf]

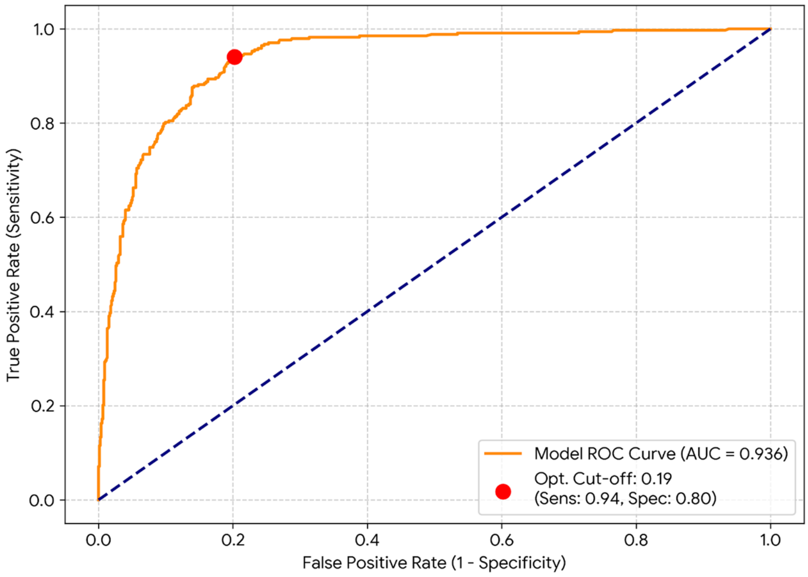

Figure S1: ROC Curve of the Firth Penalized Multivariable Model

Table S1. Relationship Between ESI Score and Emergency Department Length of Stay

	**ESI-1**	**ESI-2**	**ESI-3**	**ESI-4**	**ESI-5**	**P value**
Length of Stay (min)	124(64.25-172	92(56-195.5)	132.5(87.75-225.25)	72.25(35-108.9)	3(2-7)	<0.001

*Data are presented as medians (IQR). ESI: Emergency Severity Index.

Table S2. Comparison of Emergency Department Length of Stay Across Triage Areas

	**Red Zone (I)**	**Yellow Zone (II)**	**Green Zone (III)**	**p value**	**I-II**	**I-III**	**II-III**
Length of Stay	118(60.5-182.5)	132.5(87.75-225.25)	7(2-30)	<0.001	>0.999	<0.001	<0.001

*Data are presented as medians (IQR). Multi-group comparisons indicate that lengths of stay in both the Red and Yellow zones are significantly longer than in the Green zone.

Table S3. Model Goodness-of-Fit and Internal Validation

Performance and Fit Criteria	**Explanatory Power**		**Model Calibration**		**Discriminative Ability**	
	McFadden R^2	Nagelkerke R^2	Apparent Brier Score	Corrected Brier Score *	Apparent AUC	Corrected AUC *
	0.496	0.645	0.095	0.100	0.936	0.929

* Internal validation and optimism-correction were performed utilizing a bootstrap resampling method with 500 repetitions to estimate the expected performance on unseen data. Mean Variance Inflation Factor (VIF) values for all predictors were strictly < 2.5, confirming the absence of multicollinearity. Abbreviations: AUC: Area Under the Curve.
